# Extensive Intraductal Component (EIC) as the Most Predictive Factor for Residual Disease Post–Breast‐Conserving Surgery With Close DCIS Margins: A Single Institutional Experience

**DOI:** 10.1155/ijbc/8785445

**Published:** 2025-12-28

**Authors:** Ibrahim Elsharawi, Gillian Bethune

**Affiliations:** ^1^ Division of Anatomical Pathology, Department of Pathology and Laboratory Medicine, Dalhousie University, Halifax, Nova Scotia, Canada, dal.ca

## Abstract

**Purpose:**

We set out to assess whether the extensive intraductal component (EIC) status in invasive breast cancers serves as an independent predictor of residual disease (RD) in re‐excisions performed at our institution. This laboratory‐based study provides insights into the thresholds for additional surgical intervention in cases with close ductal carcinoma in situ (DCIS) margins following initial breast‐conserving surgery (BCS). We also examined the unique characteristics specific to EIC‐positive cases.

**Methods:**

BCS cases with invasive breast cancer and DCIS with close margins that had re‐excisions following initial surgery (Dec 2019–Dec 2024) were selected and classified into EIC positive or EIC negative. Data collected on the initial excision included the EIC status and other clinicopathological information such as margin status, DCIS extent, cancer type and focality, TNM stage, biomarker status, and OncotypeDX Recurrence Score (RS). The RD status was collected on re‐excision specimens.

**Results:**

Ninety‐one cases were included (57 EIC positive and 34 EIC negative), with most being invasive ductal carcinoma. The rate of RD on re‐excision was 70.2% and 32.4% in EIC‐positive and EIC‐negative cases, respectively (*p* < 0.001). EIC‐positive cases showed a higher tendency to involve multiple margins, had a lower T stage and greater DCIS extent, and they were more commonly associated with multifocal cancer. Finally, when assessing predictors of RD, EIC status emerged as the most significant factor among other variables (adjusted odds ratio = 3.39). Secondary findings included a relatively increased proportion of EIC‐positive cases (19%) exhibiting mucinous morphology (*p* = 0.0063) and HER2‐positive tumor status (*p* = 0.035).

**Conclusion:**

Findings show that EIC status is the most significant predictor of RD following BCSs with close DCIS margins. This emphasizes the importance of identifying EIC‐positive cases in pathology reports and prioritizing them for additional re‐excision when DCIS margins are close.

## 1. Introduction

The extensive intraductal component (EIC) status is an important element of a breast cancer resection pathology report [1]. It is defined as ductal carcinoma in situ (DCIS) involving ≥ 25% of the tumor and extending beyond the confines of the tumor [1]. Occurring in up to 30% of early stage cancers, EIC has been shown to be a predictor of residual disease (RD) and locoregional recurrence following breast‐conserving surgery (BCS), though reported incidences vary [2–4].

BCS is a standard treatment for local control of breast cancer [3, 5]. Surgeons aim to achieve negative margins, typically determined by gross evaluation or imaging guidance [3]. Nevertheless, up to 60% of cases may require re‐excision, imposing strain on the healthcare system, increasing costs, and causing inconvenience to patients [5, 6]. Some studies have highlighted that re‐excisions often occur even when margins are negative, out of an abundance of caution [7]. Given the availability of multimodal treatment options, surgeons must weigh the decision to pursue re‐excision versus alternative therapies such as radiation [7]. The decision to re‐excise involves consideration of tumor characteristics, patient preferences, and institutional resources. Margin status, in particular, plays a central role. The definition of a clear surgical margin has been a topic of ongoing debate [8]. The Society of Surgical Oncology, the American Society for Radiation Oncology, and the American Society of Clinical Oncology recommend that “tumor on ink” defines a positive margin in invasive carcinoma, while a 2 mm margin is advised for DCIS [7, 9]. However, RD has been observed even in the absence of tumor on ink, highlighting the influence of other variables such as tumor grade and size [5]. Identifying a single predictor of RD remains challenging and typically requires a multifactorial approach.

In this laboratory‐based study at a tertiary care center, we set out to determine the likelihood of RD following BCS in breast cancer cases with close DCIS margins (≤ 2 mm), stratifying them by EIC‐positive (EIC‐pos) and EIC‐negative (EIC‐neg) status. We evaluated the interplay between EIC status and other predictive variables (e.g., margin status and DCIS extent) using both univariate and multivariate analyses. We also highlight trends unique to EIC‐pos cases in our cohort. While most prior studies assessing the relationship between EIC and RD used older data, our cohort generally adhered to contemporary surgical margin guidelines and rigorous pathology documentation standards [3].

## 2. Materials and Methods

The study was approved and conducted in accordance with the Nova Scotia Health (NSH) research ethics board (REB). A search was conducted using the laboratory information system for all BCS cases (December 2019–December 2024) that showed invasive carcinoma with associated DCIS and the following additional inclusion criteria: (1) DCIS with a close margin (≤ 2 mm) and (2) re‐excision after initial surgery (within 3–6 months). Cases that received neoadjuvant therapy and did not undergo re‐excision were excluded.

For every case, the following information was collected from the initial surgery: the EIC status (EIC‐pos vs. EIC‐neg), number/location of close DCIS margins involved, DCIS extent (in millimeter), DCIS grade (highest), invasive cancer margin status (involved or not), cancer type, cancer focality (uni‐ or multifocal), TNM stage, lymphovascular invasion (LVI) status, biomarker status, and OncotypeDX RS (if available). For the re‐excision specimens, we identified the presence or absence of RD and the form of RD (DCIS only or DCIS + invasive carcinoma). Cases in which pathologists requested additional sections after the initial histopathology assessment were also documented.

Biomarker testing (ER, PR, and HER2) was performed in‐house using immunohistochemistry for all cases according to ASCO‐CAP guidelines. Eight EIC‐pos cases did not have a HER2 status available. OncotypeDX RSs (Exact Sciences, Madison, Wisconsin) were available for only an eligible subset of the study population (*n* = 33; 18 in the EIC‐pos group and 15 in the EIC‐neg group).

The clinical characteristics relating to EIC status and RD were compared using chi‐square tests for categorical variables and *t*‐tests or Kruskal–Wallis tests for continuous variables. EIC status, DCIS margin involvement, and DCIS extent were the variables used in multivariable logistic regression models based on their hypothesized role in discriminating patients at increased risk of RD. All statistical analyses were performed using StataNow 18.5 (StataCorp LLC, College Station, Texas). ChatGPT was used solely for grammatical editing.

## 3. Results and Discussion

### 3.1. Initial Excisions

A total of 91 cases with BCSs were identified, subclassified into 57 EIC‐pos and 34 EIC‐neg. Most cases had wire localization excisions (71 total, 47 EIC‐pos and 24 EIC‐neg), and the remaining were lumpectomies. The EIC‐pos cases accounted for a larger share of the cases included in our search compared to EIC‐neg (63% vs. 37%) suggesting that they constitute a higher proportion of cases with close DCIS margins that undergo re‐excisions.

The characteristics of both groups including their RD outcomes are presented in Table 1. Most cases were invasive ductal carcinoma (IDC) (EIC‐pos: 43/57 [75%]; EIC‐neg: 34/34 [100%]). Eleven EIC‐pos cases (19%) exhibited mucinous morphology, either focally or entirely; two cases were invasive lobular carcinoma, and one was micropapillary carcinoma. Both groups included one case each of mixed invasive ductal and lobular carcinoma, which was categorized under IDC. One of the mucinous cases in the EIC‐pos group was classified as mucinous micropapillary carcinoma.

**Table 1:** Characteristics of EIC‐pos and EIC‐neg cases on initial resection.

	**EIC negative** **N** = 34	**EIC positive** **N** = 57	**p** **value**
# of margins involved			< 0.001
1	24 (70.6%)	15 (26.3%)	
2	8 (23.5%)	21 (36.8%)	
3	2 (5.9%)	14 (24.6%)	
5	0 (0.0%)	3 (5.3%)	
6	0 (0.0%)	4 (7.0%)	
At least two margins (initial resection)	10 (29.4%)	42 (73.7%)	< 0.001
DCIS margin status			0.34
2 mm	4 (11.8%)	2 (3.5%)	
≤ 1 mm	15 (44.1%)	26 (45.6%)	
Involved	15 (44.1%)	29 (50.9%)	
Positive invasive cancer margin	13 (38.2%)	13 (22.8%)	0.15
DCIS grade			0.79
1	3 (8.8%)	4 (7.0%)	
2	22 (64.7%)	34 (59.6%)	
3	9 (26.5%)	19 (33.3%)	
DCIS extent (mm)	23.6 (20.4)	45.0 (22.1)	< 0.001
Multifocal invasive cancer	7 (20.6%)	24 (42.1%)	0.036
Biomarker			0.035
ER/PR+/HER2−	25 (73.5%)	37 (75.5%)	
ER/PR+/HER2+	4 (11.8%)	2 (4.1%)	
ER/PR−/HER2+	0 (0.0%)	7 (14.3%)	
ER/PR‐HER2−	5 (14.7%)	3 (6.1%)	
T stage			0.003
1mi	0 (0.0%)	13 (22.8%)	
1a	2 (5.9%)	10 (17.5%)	
1b	7 (20.6%)	9 (15.8%)	
1c	12 (35.3%)	18 (31.6%)	
T2	11 (32.4%)	7 (12.3%)	
T3	2 (5.9%)	0 (0.0%)	
LN status			0.16
NX/0	21 (61.8%)	44 (77.2%)	
1mi	3 (8.8%)	5 (8.8%)	
1	10 (29.4%)	7 (12.3%)	
2	0 (0.0%)	1 (1.8%)	
Lymphovascular invasion	14 (41.2%)	16 (28.1%)	0.25
OncotypeDX score ≥ 25	4/15 (26.7%)	2/18 (11.1%)	0.37
Residual disease (re‐excision)	11 (32.4%)	40 (70.2%)	< 0.001

In the initial excisions, the DCIS margins for all cases were ≤ 1 mm or involved, except for six cases that had margins of 2 mm (two EIC‐pos and four EIC‐neg). EIC‐pos cases had a significantly higher likelihood of involving multiple DCIS margins (≥ 2) on the initial resection compared to the EIC‐neg group (42/57 vs. 10/34, *p* < 0.001), consistent with findings reported in the literature [2]. This observation may reflect the difficulties encountered from radiological and surgical perspectives in achieving clear margins in EIC‐pos cases. Literature has emphasized the importance of employing multiple imaging modalities, such as magnetic resonance and ultrasound, in conjunction with wider excisions to achieve better margin control in EIC‐pos cases [3]. The mean extent of DCIS was 45 mm in the EIC‐pos group and 23.6 mm in the EIC‐neg group (*p* < 0.001). Positive invasive carcinoma margins were observed in 13 of 57 (22.8%) EIC‐pos cases and 13 of 34 (38.2%) EIC‐neg cases, respectively. This difference suggests that re‐excisions for EIC‐neg cases may be in part driven by invasive carcinoma margins rather than DCIS margins.

### 3.2. Re‐Excisions

**Figure 1:**
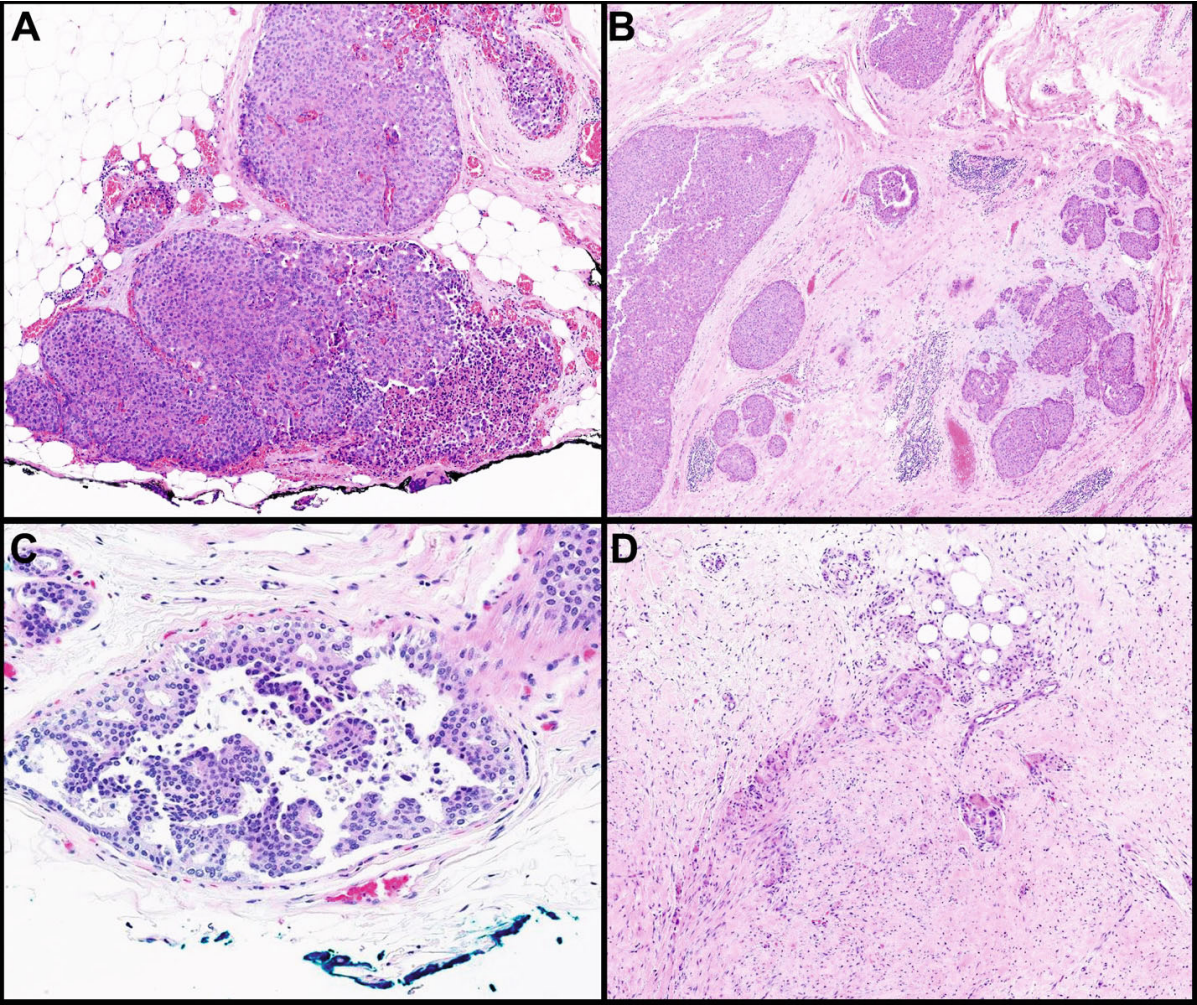
Example of an (A, B) EIC‐pos and (C, D) EIC‐neg cases. (A, B) EIC‐pos case: (A) an EIC‐pos case with a positive posterior margin involvement by DCIS (H&E, 8×). (B) The residual disease in the re‐excision of that case with both DCIS and microinvasive carcinoma foci (H&E, 4×). (C, D) An EIC‐negative case with (C) a close DCIS margin (< 1 mm) on the initial resection (H&E, 8×) and (D) a re‐excision showing postsurgical changes with no residual disease (H&E, 4×).

The rate of RD on re‐excision was 70.2% (40/57) in EIC‐pos cases and 32.4% (11/34) in EIC‐neg cases (*p* < 0.001) (Table 1). Figure 1 demonstrates an example of an EIC‐pos case with involved margins on initial resection (Figure 1A) and RD on re‐excision (Figure 1B) as well as an EIC‐neg case with close DCIS margins (< 1 mm) (Figure 1C) and no RD on re‐excision (Figure 1D). Among the 40 EIC‐pos cases with RD, 25 (62.5%) exhibited DCIS exclusively, while the remaining 15 cases had both DCIS and invasive carcinoma. In contrast, the 11 EIC‐neg cases with RD were nearly equally divided between DCIS only (five cases) and DCIS with invasive carcinoma (six cases). This difference was not statistically significant (*p* = 0.309). Of the 15 EIC‐pos cases with invasive carcinoma on re‐excision, eight (53%) had clear invasive margins on initial resection, suggesting that the invasive carcinoma in the re‐excision may represent multifocal disease. These findings emphasize the importance of re‐excision for close DCIS margins to evaluate for residual invasive disease despite the invasive carcinoma margin status. Holland et al. reported that EIC‐pos breast cancers had a significantly greater likelihood of an intraductal component extending more than 2 cm from the primary tumor, compared to EIC‐neg cases [10].

Table 2 summarizes the characteristics of the cases with and without RD on re‐excision. As expected, a significant portion of the RD group was EIC‐pos (78.4%, *p* < 0.001). Another influential factor was the extent of DCIS. In an unadjusted logistic regression analysis, EIC status and DCIS extent were associated with increased odds of RD (Table 3). Model 1, which adjusted for EIC status, DCIS extent, and margin status, showed that the presence of at least two margins was no longer significantly associated with odds of RD after adjusting for EIC positivity and DCIS extent. It was therefore removed from Model 2, which found that EIC positivity remained strongly associated with RD (OR: 3.26; 95% CI: 1.20–8.87), while DCIS extent showed a modest association (OR: 1.02; 95% CI: 1.00–1.05). These findings highlight the importance of reporting EIC status in breast cancer excision specimens.

**Table 2:** Characteristics of cases with and without residual disease.

	**No residual disease** **N** = 40	**Residual disease** **N** = 51	**p** **value**
EIC positive	17 (42.5%)	40 (78.4%)	< 0.001
# of margins involved			0.28
1	21 (52.5%)	18 (35.3%)	
2	12 (30.0%)	17 (33.3%)	
3	6 (15.0%)	10 (19.6%)	
5	1 (2.5%)	2 (3.9%)	
6	0 (0.0%)	4 (7.8%)	
At least two margins (initial resection)	19 (47.5%)	33 (64.7%)	0.14
DCIS margin status			0.26
2 mm	4 (10.0%)	2 (3.9%)	
≤ 1 mm	20 (50.0%)	21 (41.2%)	
Involved	16 (40.0%)	28 (54.9%)	
Positive invasive cancer margin	11 (27.5%)	15 (29.4%)	1.00
DCIS grade			0.50
1	4 (10.0%)	3 (5.9%)	
2	26 (65.0%)	30 (58.8%)	
3	10 (25.0%)	18 (35.3%)	
DCIS extent (mm)	27.9 (21.5)	44.1 (23.2)	< 0.001
Multifocal invasive cancer	11 (27.5%)	20 (39.2%)	0.24
Biomarker			0.010
ER/PR+/HER2−	26 (72.2%)	36 (76.6%)	
ER/PR+/HER2+	5 (13.9%)	1 (2.1%)	
ER/PR−/HER2+	0 (0.0%)	7 (14.9%)	
ER/PR‐HER2−	5 (13.9%)	3 (6.4%)	
T stage			0.32
1mi	3 (7.5%)	10 (19.6%)	
1a	7 (17.5%)	5 (9.8%)	
1b	9 (22.5%)	7 (13.7%)	
1c	13 (32.5%)	17 (33.3%)	
T2	8 (20.0%)	10 (19.6%)	
T3	0 (0.0%)	2 (3.9%)	
LN status			0.35
NX/o	32 (80.0%)	33 (64.7%)	
1mi	2 (5.0%)	6 (11.8%)	
1	6 (15.0%)	11 (21.6%)	
2	0 (0.0%)	1 (2.0%)	
Lymphovascular invasion	10 (25.0%)	20 (39.2%)	0.18
OncotypeDX score ≥ 25	1 (9.1%)	5 (22.7%)	0.64

**Table 3:** Unadjusted and adjusted associations with odds of residual disease.

**Variable**	**Unadjusted OR**	**95% CI**	**Model 1: Adjusted OR**	**95% CI**	**Model 2: Adjusted OR**	**95% CI**
EIC positive	4.92	1.97, 12.3	3.39	1.16, 9.92	3.26	1.20, 8.87
DCIS extent (mm)	2.03	0.87, 4.72	0.89	0.32, 2.52	1.02	1.00, 1.05
≥ 2 margins	1.03	1.01, 1.06	1.02	1.00, 1.05	—	—

*Note:* Model 1 includes all three variables; Model 2 includes EIC positivity and DCIS extent.

Abbreviations: CI, confidence interval; OR, odds ratio.

Although the recommendation for a positive invasive carcinoma margin (defined as tumor on ink) would appear to be a more reliable predictor of RD amidst the debate surrounding the 2 mm cutoff for DCIS margins, our research shows that positive invasive carcinoma margins do not significantly affect the likelihood of RD on re‐excisions (Table 2) [7, 11, 12]. This emphasizes the critical role of DCIS margins and EIC status in determining the necessity for re‐excision. Nonetheless, our results might be biased due to case selection in the study, which was based on close DCIS margins without regard to the invasive carcinoma margins.

Despite the lack of statistical significance, the rate of RD was higher in cases with two or more close DCIS margins. Some literature has shown that the volume of disease at the margin influences the likelihood of RD [1, 13, 14]. While it is not a mandatory reporting element to measure the extent of DCIS close to the margin, it may be valuable to further investigate the relationship between the DCIS extent at the close margin and the risk of RD while also incorporating the EIC status.

### 3.3. Additional Findings

#### 3.3.1. Clinicopathological Characteristics

**Figure 2:**
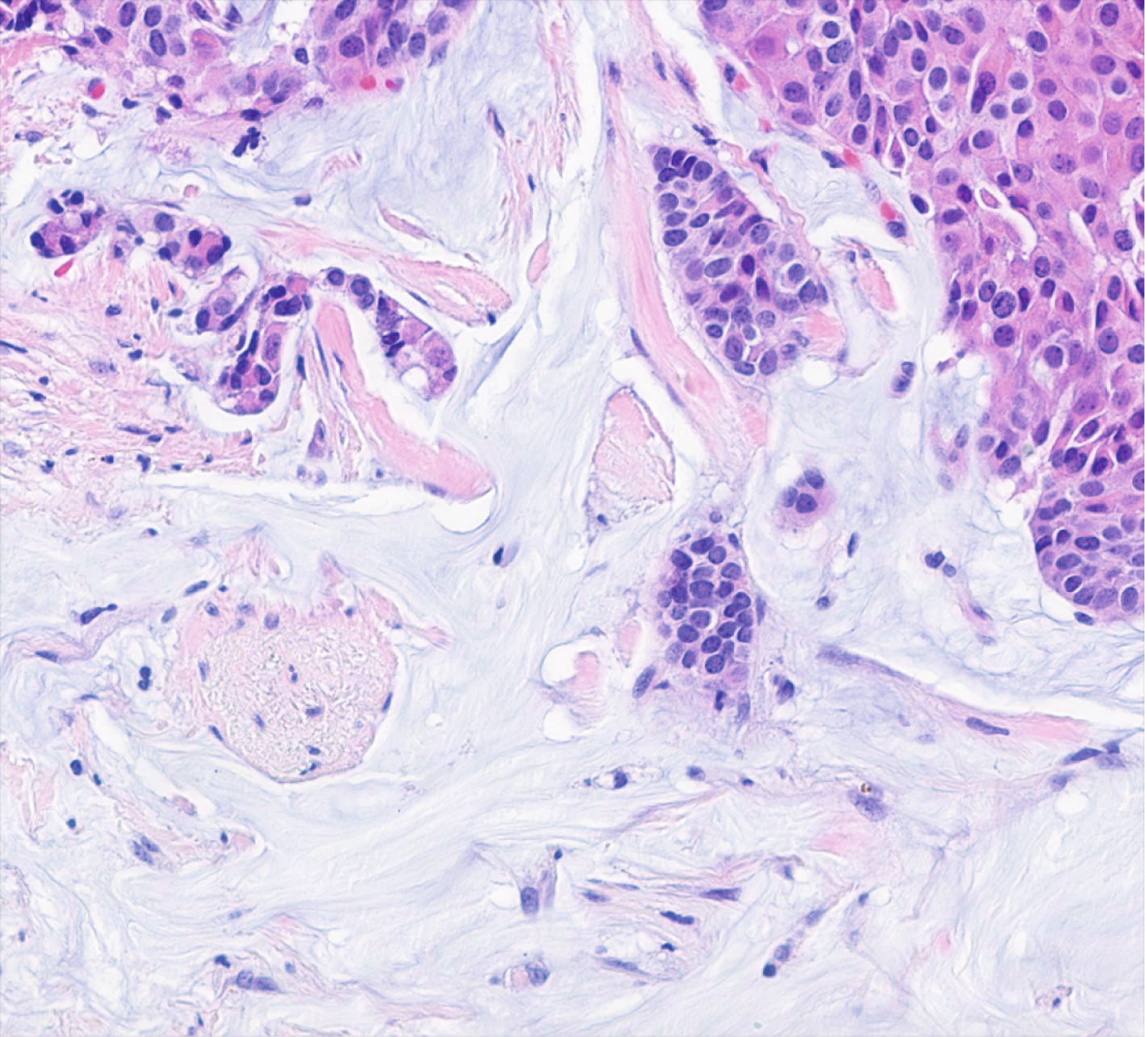
A representative case of EIC‐positive mucinous breast cancer. H&E section of the case showing clusters of neoplastic cells embedded in pools of mucin (20×).

The EIC‐pos group showed a tendency to exhibit mucinous morphology (11/57 vs. 0/34, *p* = 0.0063). A representative case of an EIC‐pos mucinous carcinoma is shown in Figure 2. The limited size of our study population however prevents us from drawing definitive conclusions. There is limited literature discussing this potential association. A study by Kitchen et al. found that 1.2% of their EIC‐neg cases were mucinous carcinomas, compared to 1.9% in the EIC‐pos group; however, only eight mucinous carcinoma cases were included in that study [2]. As EIC is commonly associated with early stage cancers, one might speculate that EIC‐pos and mucinous breast cancers share a common characteristic of having a favorable prognosis [15–17]. Nonetheless, larger studies are warranted to investigate this potential association.

EIC‐pos cases were also more frequently associated with lower T‐stage disease (*p* = 0.003) and multifocal cancer (42.1% vs. 20.6%, *p* = 0.036). The biomarker profile varied slightly between the two groups (*p* = 0.035), with a relatively higher proportion of HER2‐positive tumors in the EIC‐pos group (nine vs. four cases). While some studies have reported an increased proportion of HER2‐positive tumors among EIC‐pos cases, further investigation is necessary to explore this potential association [18].

No clear trends were observed regarding the relationship between EIC status and lymph node involvement, DCIS grade, or LVI. With regard to the OncotypeDX RS, the proportion of cases with a high‐risk RS (≥ 25) was lower in the EIC‐pos group compared to the EIC‐neg group (11.1%, 2/18 vs. 26.7%, 4/15). Additionally, the mean RS differed between groups (EIC‐pos: 16.7 vs. EIC‐neg: 22.1, *p* = 0.042). Recent studies have noted a correlation between lower RS values and EIC positivity, indicating that the distant recurrences are less frequent in EIC‐pos cases despite their increased propensity for RD and local recurrences [2, 3, 19].

#### 3.3.2. Other Factors Impacting RD

As previously stated, the main statistically significant factors impacting RD are the EIC status and DCIS extent. Variables such as the highest DCIS grade, multifocal cancer status, TN stage, LVI, and OncotypeDX RS did not appear to significantly impact the likelihood of finding RD on re‐excision. While evaluating the relationship between biomarker status and RD is challenging—given that most cases were ER/PR‐positive and HER2‐negative—HER2‐positive cases appeared to be slightly overrepresented in the group with RD, likely due to their association with EIC‐pos cases, as previously described. Ohri et al. showed that HER2‐positive EIC‐pos cases were more likely to be associated with RD following BCS [18].

Previous studies examining the risk factors associated with RD post‐BCSs have identified several key variables including the size (invasive and DCIS), the number of margins involved, and the nodal stage [5, 6, 20]. Our study however highlights that the odds of RD are significantly impacted by the EIC status, particularly when compared to previously identified risk factors (e.g., T stage). We acknowledge that a comprehensive approach accounting for multiple variables is essential to assess the risk of RD and the need for re‐excision following BCS.

## 4. Role of Pathologic Sampling for EIC Determination

From a surgical pathology laboratory perspective, the implementation of standardized procedures for specimen orientation (e.g., by sutures), along with established protocols for inking and margin documentation, has significantly enhanced breast pathology practice and patient care overall. Some studies have highlighted that intraoperative inking is superior for predicting RD and determining the necessity for re‐excisions compared to post‐suture orientation inking [21, 22]. Wire or magnetic seed–guided localization excisions have also added value in localizing nonpalpable lesions and DCIS [23]. Based on our local practice, BCSs may be submitted in total (if small) or limited to representative sections if large. Diagrammatic or photographic mapping of sections is critical for proper evaluation of DCIS extent and for establishing the EIC status in relation to an invasive carcinoma. In addition, adequate sampling is crucial for evaluating margin status. Eleven cases in our study required additional submission of sections at the pathologists′ discretion: seven EIC‐pos cases (12.2%) and four EIC‐neg cases (11.7%). The intent of additional sampling in these cases was to confirm margin status, and it also clarified the extent of DCIS in at least two of the EIC‐pos cases. Six of the seven EIC‐pos cases that underwent additional sampling showed RD, while only one of four EIC‐neg cases showed RD on re‐excision. Given that EIC status is the most significant variable in predicting RD in cases with close DCIS margins, thorough gross and histopathological evaluation is required to confidently determine EIC status.

## 5. Limitations

The relatively small sample size in this study can be attributed to several factors. Primarily, we concentrated on a specific subset of breast cancer cases (i.e., cases of associated DCIS with close margins regardless of invasive cancer margin status, those with re‐excisions only, and excluding patients who received neoadjuvant therapy). In addition, the number of re‐excisions may have been significantly impacted by the COVID‐19 pandemic, which coincided with our research period. Although we intended to include cases of DCIS with margins greater than 2 mm to better understand the impact of EIC status, the number of cases with re‐excisions in this group was too small for inclusion in our analysis.

## 6. Conclusion

Our findings confirm that the EIC status can independently predict RD following BCS. EIC‐pos cases are more frequently associated with multiple margin involvement, greater DCIS extent, multifocal invasive carcinoma, and a lower T stage at the time of initial resection. Additional trends include an increased tendency for EIC‐pos cases to be HER2‐positive, to exhibit mucinous morphology, and to have lower OncotypeDX RSs.

## Conflicts of Interest

The authors declare no conflicts of interest.

## Funding

No funding was received for this manuscript.

## Data Availability

Data is available under reasonable request.
